# Unresectable Malignant Biliary Obstruction: Treatment by Self-Expandable Biliary Endoprostheses

**DOI:** 10.1155/1993/78590

**Published:** 1993

**Authors:** Andreas Glättli, Steven C. Stain, Hans U. Baer, Walter Schweizer, Jürgen Triller, Leslie H. Blumgart

**Affiliations:** 1 The Clinic of Visceral and Transplantation Surgery Inselspital University of Bern Bern Switzerland; 2 The Institute for Diagnostic Radiology Inselspital University of Bern Bern Switzerland; 3 Memorial Sloan-Kettering Cancer Center Department of Surgery New York USA

## Abstract

The primary goal in the treatment of malignant obstruction is the relief of jaundice. Although operative biliary bypass is a reliable method of palliation, nonoperative palliation may be desirable in selected patients.

We report our experience with forty-eight self expandable metallic biliary endoprostheses (Wallstent) percutaneously placed in 35 patients with irresectable malignant biliary obstruction. In twelve patients more than one stent was necessary to bridge the entire length of the biliary stenosis. The obstruction was due to primary tumors in 14 and to lymph node metastases in 12. In nine patients transanastomotic stents were placed after previous bilioenteric anastomosis because of malignant obstruction. Complications occurred in 11 patients (31.4%), and five patients died within 30 days of stent placement (14.3%). The mean stent patency to date of patients discharged is 6.1 months, and the mean survival 7.2 months. Follow up data is available for 29 patients, and excellent palliation was achieved for more than 75% of the survival time in 22 (76%). Seven patients have had documented stent occlusion requiring further intervention (24%).

In this selected group of patients, the results of percutaneous self-expandable stents are encouraging. However, our data does not support the initial reports of self-expandable endoprostheses that suggest an improved result compared to conventional plastic stents. A randomized study using either expandable stents as compared to operative biliary enteric bypass is necessary.


HPB Surgery, 1993, Vol. 6, pp. 175–184
Reprints available directly from the publisher
Photocopying permitted by license only
© 1993 Harwood Academic Publishers GmbH
Printed in the United States of America

### UNRESECTABLE MALIGNANT BILIARY OBSTRUCTION: TREATMENT BY SELF-EXPANDABLE BILIARY ENDOPROSTHESES

ANDREAS GLÄTTLI[1], STEVEN C. STAIN[1], HANS U. BAER[1], WALTER SCHWEIZER[1], JÜRGEN TRILLER[2] and LESLIE H. BLUMGART[3]

*[1]The Clinic of Visceral and Transplantation Surgery and [2]The Institute for Diagnostic Radiology, Inselspital, University of Bern, Bern, Switzerland and [3]Memorial Sloan-Kettering Cancer Center, Department of Surgery, New York*

*(Received 30 March 1992)*

The primary goal in the treatment of malignant obstruction is the relief of jaundice. Although operative biliary bypass is a reliable method of palliation, nonoperative palliation may be desirable in selected patients.

We report our experience with forty-eight self expandable metallic biliary endoprostheses (Wallstent) percutaneously placed in 35 patients with irresectable malignant biliary obstruction. In twelve patients more than one stent was necessary to bridge the entire length of the biliary stenosis. The obstruction was due to primary tumors in 14 and to lymph node metastases in 12. In nine patients transanastomotic stents were placed after previous bilioenteric anastomosis because of malignant obstruction. Complications occurred in 11 patients (31.4%), and five patients died within 30 days of stent placement (14.3%). The mean stent patency to date of patients discharged is 6.1 months, and the mean survival 7.2 months. Follow up data is available for 29 patients, and excellent palliation was achieved for more than 75% of the survival time in 22 (76%). Seven patients have had documented stent occlusion requiring further intervention (24%).

In this selected group of patients, the results of percutaneous self-expandable stents are encouraging. However, our data does not support the initial reports of self-expandable endoprostheses that suggest an improved result compared to conventional plastic stents. A randomized study using either expandable stents as compared to operative biliary enteric bypass is necessary.

KEY WORDS: Bile-duct obstruction, malignancy, jaundice, palliaton, expandable endoprosthesis

### INTRODUCTION

Malignant biliary obstruction may be treated by operative or nonoperative methods. Operative resection should be performed when possible and provides relief of biliary obstruction, with a chance of cure. However the majority of patients with either primary or metastatic tumors are not amenable to curative resection[1,2]. Palliative relief of jaundice may be achieved by operative biliary bypass, or transtumoral stenting[3,4,5,6]. Although operative bypass provides a reliable method of palliation[4,5], it is not always possible, and the relatively short life expectancy of these patients suggests a less invasive alternative may be preferable if the method is safe, effective, and does not require further intervention.

Address correspondence to: Andreas Glättli, M.D., Clinic for Visceral and Transplantation Surgery, Inselspital, University of Bern, CH-3010 Bern, Switzerland.

Permanent stainless steel biliary endoprostheses have been developed, and preliminary reports suggest improved results compared to conventional plastic stents[7–13]. The reported advantages include safety of insertion, and a lower incidence of stent occlusion and subsequent cholangitis[11,12]. This study describes our initial experience with percutaneous placement of self-expandable biliary endoprostheses (Wallstent) for irresectable malignant biliary obstruction.

### MATERIALS AND METHODS

Between December 1988 and June 1991, 48 expandable biliary endoprostheses were placed in 35 patients with malignant biliary obstruction. There were 16 males and 19 females, with a mean age of 61.7 years (range 34–84). A single stent was used in 23 patients. Eleven patients required two stents each, and one patient had three stents placed. More than one stent was required in these patients to bridge the entire length of the biliary stenosis. Twenty six of the 35 patients (74%) had histologic proof of malignancy prior to stent placement, and malignancy was evident by the clinical course of the remaining nine. The obstruction was due to a primary tumor in 14 patients, and lymph node metastases in 12 (Table 1). In nine patients the stents were placed for biliary obstruction after previous bilioenteric anastomosis. Four of these patients had a history of resection and bypass of a tumor originating from the gallbladder, pancreas or bile duct. Five patients had previously been treated by a palliative bypass for irresectable cholangiocarcinoma. The locations of the biliary obstruction were defined as: high (confluence, main hepatic duct -- 11), middle (common hepatic duct, supraduodenal common bile duct — 10), distal (intrapancreatic — 5), and transanastomotic (after previous bilioenteric anastomosis — 9).

In our institution we have pursued an aggressive policy of exploration for possible resection of all patients with biliary and pancreatic tumors without radiologically demonstrable contraindication to resection. Operative bypass is selected in fit patients who are believed to be irresectable, if a suitable hepatic duct

**Table 1**

| *Etiology of Obstruction* | | *(N)* |
|---|---|---|
| Primary tumor | | 14 |
| cholangiocarcinoma | 6 | |
| gallbladder | 5 | |
| pancreas | 3 | |
| Lymph node metastases | | 12 |
| stomach | 5 | |
| colon | 4 | |
| breast | 2 | |
| leiomyosarcoma | 1 | |
| Previous bilioenteric anastomosis | | |
| cholangiocarcinoma | 7 | 9 |
| pancreas | 1 | |
| gallbladder | 1 | |

is available and when lobar liver atrophy is not present. Most patients reported in this series were considered inoperable because of poor medical condition, or when bile duct anatomy was not favorable for operation.

The decision to perform palliative drainage by the percutaneous route was made at a multidisciplinary meeting involving surgeons, interventional radiologists and gastroenterologists. The length of hospitalization included time for diagnostic studies to determine resectability. All patients were managed on the surgical ward, and routine follow up visits were done in the surgical outpatient clinic. Repeat radiologic investigations were performed only if clinical symptoms suggested stent occlusion. Early and late complications were documented, as well as the necessity of repeat admissions or interventions. The serum bilirubin and alkaline phosphatase levels were measured at admission and compared to the level at discharge and three months after stent placement.

### TECHNIQUE OF INSERTION

All biliary interventions were performed after administration of prophylactic antibiotics (Piperacillin). The endoprostheses were placed in two stages. A percutaneous transhepatic biliary drainage was first performed under local anesthesia. The stent insertion was performed at a second session with local anesthesia, intravenous sedation and cardiovascular monitoring. Infrequently, general anesthesia was required.

An ultra thin guide wire (.018″) was passed through a Chiba needle and advanced into the common bile duct. The introducer set (Accustic-set, Boston Scientific) was passed over a guide wire beyond the point of obstruction. The introducer was exchanged for an 8.3 F Ring drainage catheter and maintained as an internal / external biliary drain for two to four days. After the temporary biliary drainage, the stent insertion was performed by first introducing a 7 F sheath. A 7 F balloon catheter (length 6 cm, diameter 10 mm) was used to dilate the stenosed bile duct. The endoprosthesis (Wallstent, Schneider, Zürich, Switzerland) mounted on the tip of a 7 F introducer catheter, was passed over a guide wire distal to the stenosis. After proper positioning of the endoprosthesis, additional balloon dilatation of the stenosis and stent was performed. A drainage catheter was then left in the proximal portion of the implanted endoprosthesis. At 24 to 48 hours post stent placement, a repeat cholangiogram was performed to document the stent position and patency (Figures 1 and 2). As the external catheter was removed, the hepatic puncture was embolized with gelfoam strips. Antibiotic prophylaxis was continued until removal of the external drainage catheter.

The transhepatic approach was used in 30 patients. Five patients had the limb of a Roux-en-Y loop subcutaneously placed at the time of a previous operative biliary enteric bypass. The stent was placed transjejunally in four of these patients, and one required a combined endoscopic-transhepatic approach. Figure 3 shows the metal stent in the expanded state.

### RESULTS

Placement of the biliary endoprosthesis was possible in 35 of the 36 patients in

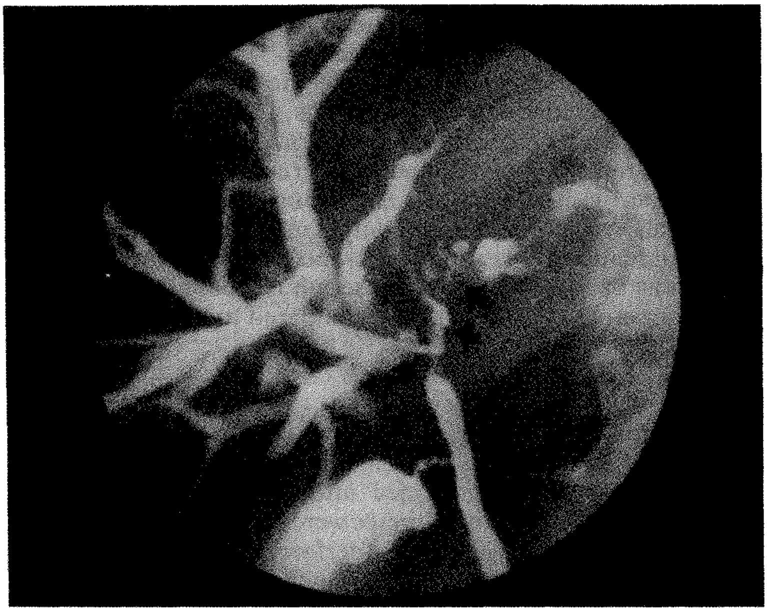

**Figure 1a** Percutaneous transhepatic cholangiogram of a hilar cholangiocarcinoma (arrows) with stenosis of right and left hepatic duct.

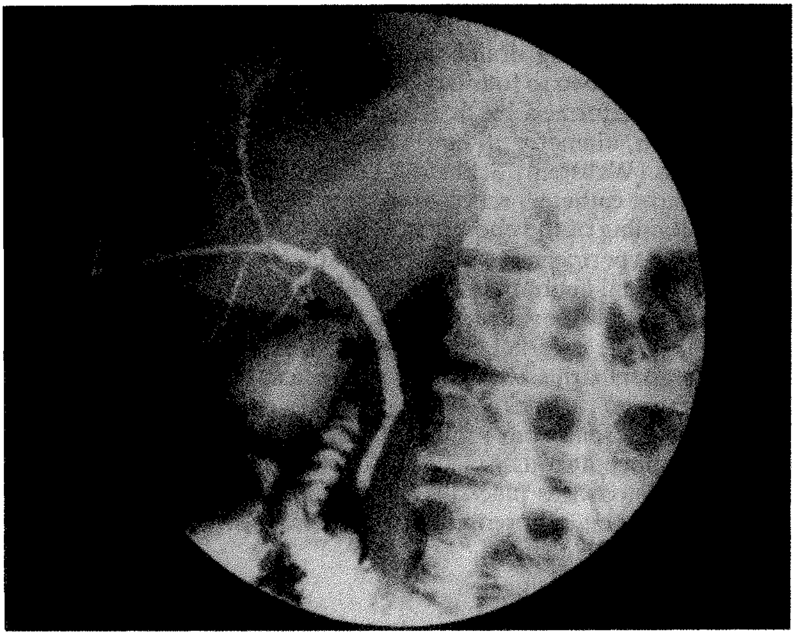

**Figure 1b** Same patient with expandable stent (arrows) placed across the stenosis into the right hepatic duct.

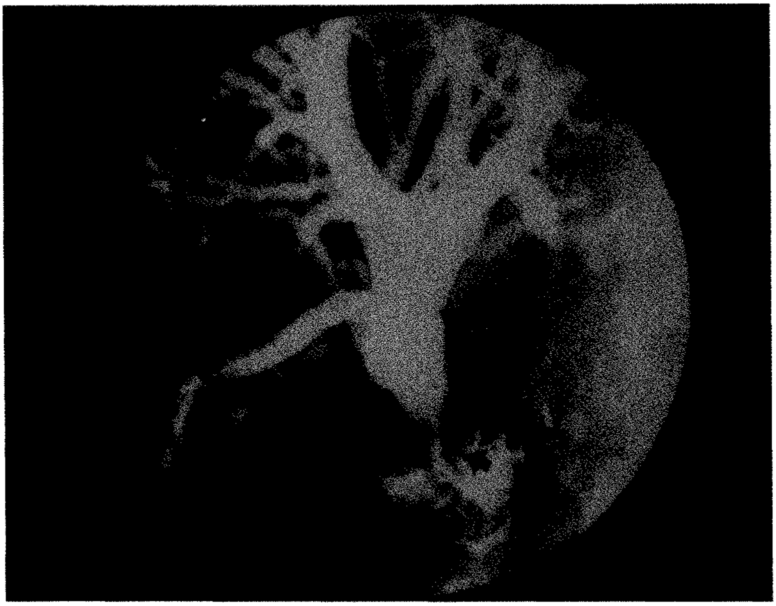

**Figure 2a** Cholangiogram shows malignant recurrence at biliary enteric anastomosis (arrows).

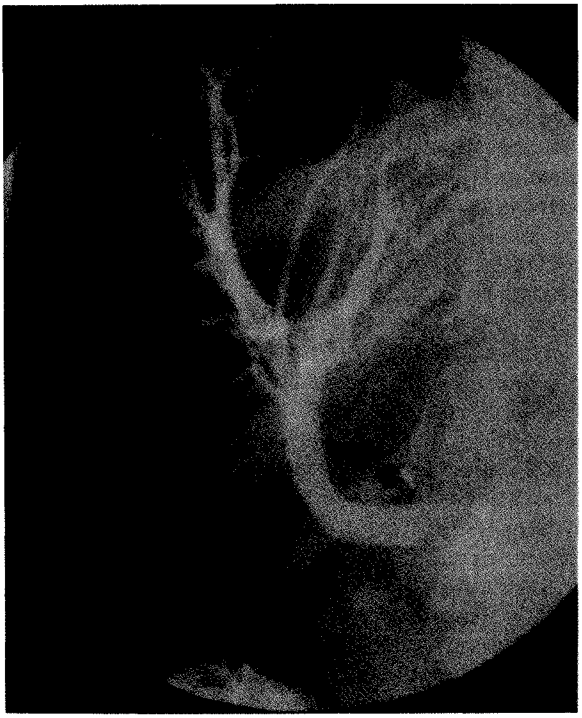

**Figure 2b** Same patient with transanastomotic stent (arrows) placed across stenosis into the jejunal loop.

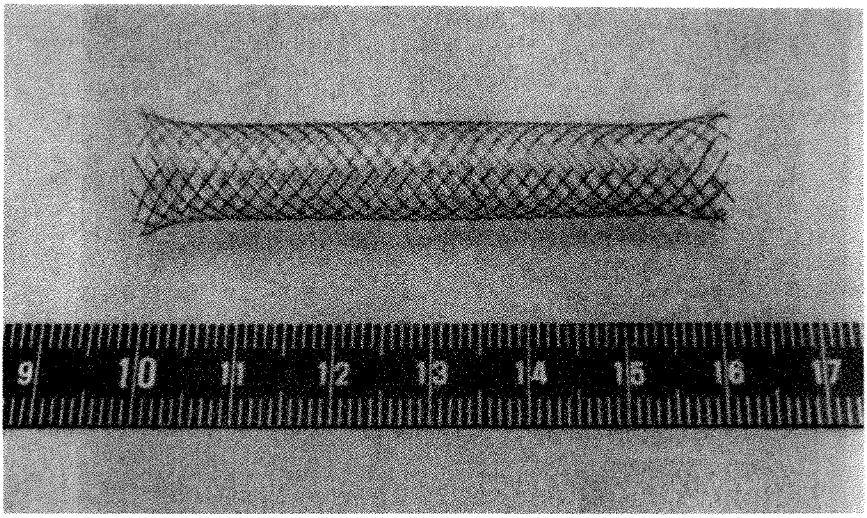

**Figure 3** Wallstent in expanded state.

whom it was attempted. One patient died after failed stent placement and is excluded from further analysis. The length of the hospitalization for stent placement was a mean of 15 days (range 2–35 days). Eleven patients (31%) developed complications within 30 days of stent placement. Cholangitis was the most frequent (four patients), followed by pancreatitis in two and pneumonia, renal insufficiency and a bleeding ulcer in one patient respectively; two complications were directly related to the stent placement (6%). A duodenal perforation due to a guide wire was managed conservatively, and a hepatic artery pseudoaneurysm was embolized one month after stent placement. Five patients died within 30 days of stent placement (14%). The deaths were due to persistent biliary sepsis in four patients, and necrotizing pancreatitis in one.

One patient has been lost to follow up. Data are available on 29 patients discharged from the hospital, and all have survived at least three months. Ten patients have died (34%) at a mean of 8.5 months after stent placement. Nineteen patients are alive (66%) after a mean of 6.6 months. The mean survival and stent patency according to etiology and level of obstruction are listed in Table 2. Excluding the terminal admission before death, 15 of 30 patients (50%) have required repeat hospitalizations for episodes of cholangitis or recurrent jaundice. Twelve patients have been admitted once, one patient twice, one patient four times, and one five times. Seven patients have had documented stent occlusion which required further intervention (24%) (Table 3). In three patients transhepatic insertion of an expandable stent through the previous one was successful. In two cases a conventional stent was endoscopically placed through the occluded self-expandable prosthesis. Simple recannulization of the stent was used as an alternative in two patients.

The quality of the palliation was estimated by the proportion of patients who

**Table 2**

| *Etiology* | *(N)* | *Mean survival (months)* | *Stent patency (months)* |
|---|---|---|---|
| Primary tumors | (14) | 5.9 (3–13) | 5.4 (3–11) |
| Metastases | (10) | 8.0 (3–15) | 6.6 (3–15) |
| Anastomotic | (5) | 8.8 (5–13) | 6.6 (4–13) |
| *Level of obstruction* | | | |
| High | (10) | 6.4 (3–13) | 5.5 (3–11) |
| Middle | (9) | 6.8 (3–15) | 5.3 (3–15) |
| Low | (5) | 8.0 (3–15) | 8.0 (3–15) |
| Anastomotic | (5) | 8.8 (5–13) | 6.6 (4–13) |
| Total | (29) | 7.2 (3–15) | 6.1 (3–15) |

a Refers to 29 patients discharged from hospital and available for follow up.

**Table 3**

*Reinterventions*

| *Cause* | *Interval* | *Treatment* |
|---|---|---|
| Jaundice | 6 months | Segment III bypass (operative) |
| Stent occlusion | 6 months | Conventional stent (endoscopic due to ascites) |
| Stent occlusion | 6 months<br>10 months<br>14 months | Transhepatic recannulization<br>Transhepatic 2nd stent<br>Transhepawtic 3rd stent |
| Stent occlusion | 8 months | Conventional stent (endoscopic-percutaneous) |
| Stent occlusion | 4 months | Transhepatic 2nd stent |
| Stent occlusion | 3 months | Transhepatic 2nd stent |
| Stent occlusion | 5 months | Transjejunal recannulization |

survived until they developed debilitating episodes of cholangitis or stent occlusion requiring hospitalization or repeat interventions. Twenty-two of 29 patients (76%) were generally well during greater than 75% of their survival after stent placement. Five patients (17%) were well 50% to 75% of the time after stent placement, and two patients (7%) had adequate palliation for less than 50% of survival time.

### DISCUSSION

A variety of options are available to relieve irresectable malignant obstruction. The selection of the specific treatment for an individual patient is multifactorial, but the relatively short life expectancy is one of the most important considerations. Biliary enteric bypass has been shown to be a reliable method of palliation. There are however, subsets of patients in whom operative bypass may be inappropriate due to lobar atrophy or nonavailability of a suitable bile duct. Some patients are not suitable for operation due to poor medical condition which also makes nonoperative relief of biliary obstruction preferable. Conventional plastic biliary stents have been associated with frequent episodes of cholangitis and stent occlusion. The incidence of documented stent occlusion depends upon the thoroughness of follow up, but in large series, has been reported at 6–23%[2,14,15]. Metal self expandable endoprostheses were adapted from their initial use as vascular stents in an effort to provide lasting relief of jaundice. Several centers have now reported experience with these stents for both benign and malignant biliary obstruction[7–13].

The expandable stents may be placed by either the endoscopic or transhepatic route. It has been shown for conventional stents that endoscopic insertion is superior to percutaneous placement in terms of 30-day mortality and success rate for relief of jaundice[16]. Endoscopic placement of expandable metal endoprostheses has not yet been done by our gastroenterologists. Percutaneously placed expandable stents have theoretical advantages compared to conventional stents. The conventional stent requires a 12–14 F hepatic puncture and results in a stent lumen of 4 mm. Despite the smaller hepatic puncture of the expandable stent (7 F), it results in a larger diameter after deployment (10 mm). Experimental work in animals has identified mucosal proliferation along the bile duct wall which grows between the metal filaments to form a new mucosal lining[17]. The incorporation of the stent into the bile duct wall has been suggested as allowing a more physiologic flow of bile and a reduction in the incidence of encrustation and subsequent cholangitis[9] as happens with conventional plastic stents.

Three models of expandable endoprostheses are currently available. The self expandable Gianturco stent (Cook, Bloomington, IN) is made of stainless steel wire. The balloon expandable Palmaz stent (Johnson and Johnson, Warren, NJ) is a lattice of stainless steel. The Wallstent (Schneider, Zürich, Switzerland) used in this series is self expandable, and is composed of braided stainless monofilaments (Figure 3). The Gianturco stent made of wires is more porous than the other models, and tumor may grow more easily between the wires. In malignant biliary obstruction, the narrow spaced braided Wallstents have been reported to have a lower rate of tumor growth between the filaments (7%) as compared to the Gianturco stent (50%)[12].

The initial reports of expandable biliary endoprostheses have been encouraging (Table 4). Comparisons between the different centers are difficult, as the patient selection, indications for placement, and the method and depth of follow up evaluation vary. Some of the series have reported few or no complications related to stent placement, and only rare episodes of subsequent stent occlusion. Although the patients we selected for stent placement were chosen by different criteria, this has not been our experience. During the same time period, our mortality for biliary enteric bypass in patients considered fit for operation was 11% (Baer *et al.*, submitted for publication), a result similar to the 14% in the current series. The incidence of perioperative complications after stent placement (31%) is also considerable. It is possible that some or all of the patients with pancreatitis or cholangitis after stent placement were due to progression of the disease and not directly from the stent placement. The mean hospital stay of 15 days included preoperative investigations to exclude resectability. In our experience, this is not a procedure for outpatient or minimal hospitalization as others have suggested[10,11]. Most importantly, by having all of the follow up care in the same clinic, seven of the

**Table 4**

| *Author (n)* | *Type stent* | *Series of expandable stents* *Complications* | *Mortality* | *Occlusion* | *Survival* |
|---|---|---|---|---|---|
| Irving (16) | Gianturco | not listed | 0% | 50% | not listed |
| Yoshioka (21) | Gianturco | not listed | 10% | 24% | 97 days |
| Coons (16) | Gianturco | not listed | 0% | 19% | not listed |
| Adam (41) | Wallstent | 12% | 7% | 5% | 105 days |
| Neuhaus (35) | Wallstent | 0% | 0% | 17% | 134 days |
| Lammer (31) | Wallstent | 8% | 7% | 13% | not listed |
| Cwikiel (16) | Wallstent | 11% | 19% | 31% | 189 |
| Glätrtli (35) | Wallstent | 31% | 14% | 24% | 218 days |

*n*: includes only malignant stenoses
Mortality: within 30 days of stent placement
Occlusion: documented occlusion reported
Survival: mean survival calculated from data within article

29 patients available for follow up have had documented stent occlusion requiring reintervention (24%), which is not less than after conventional stenting. We did not identify any difference in survival or stent patency based on etiology or level of biliary obstruction. We have been especially encouraged by the added months of palliation in patients with anastomotic recurrence months to years after a prior resection of a malignant tumor.

Self expandable biliary endoprostheses have an important role in the treatment of irresectable malignant biliary obstruction. A randomized study of these patients comparing expandable stents with operative biliary enteric bypass is necessary to define the role of each option. The evaluation of endoscopic insertion of self expandable metal endoprostheses should be part of such a study. Selection of patients for palliative stent placement should include consultation with members of different specialities to prevent potentially resectable patients from being denied an attempt at curative resection and to choose the optimal palliative procedure.

### *References*

1. Blumgart, L.H., Benjamin, I.S., Hadjis, N.S. and Beazley, R. (1984) Surgical approaches to cholangiocarcinoma at the confluence of hepatic ducts. *Lancet*, **i**, 66–70
2. Moossa, A.R. (1982) Pancreatic cancer — approach to diagnosis, selection for surgery and choice of operation. *Cancer*, **50**, 2689–2698
3. Gibson, R.N., Yeung, E., Hadjis, N., Adam, A., Benjamin, I.S., Allison, D.J. and Blumgart, L.H. (1988) Percutaneous transhepatic endoprostheses for hilar cholangiocarcinoma. *Am. J. Surg.*, **156**, 363–367
4. Blumgart, L.H. and Kelley, C.J. (1984) Hepaticojejunostomy in benign and malignant high bile duct stricture: approaches to the left hepatic ducts. *Br. J. Surg.*, **71**, 257–261
5. Bismuth, H. and Corlette, B.M. (1975) Intrahepatic cholangioenteric anastomosis in carcinoma of the hilus of the liver. *Surg. Gynecol. Obstet.*, **140**, 170–178
6. Terblanche, J., Kahn, D., Bornmann, P.C. and Werner, D. (1988) The role of U tube palliative treatment in high bile duct carcinoma. *Surgery*, **103**, 624–632
7. Irving, J.D., Adam, A., Dick, R., Dondelinger, R.F., Lunderquist, A. and Roche, A. (1989) Gianturco expandable metallic biliary stents: Results of an European clinical trial. *Radiology*, **172**, 321–326

8. Yoshioka, T., Sakaguchi, H., Yoshimura, H., Tamada, T., Ohishi, H., Uchida, H. and Wallace, S. (1990) Expandable metallic biliary endoprostheses: Preliminary clinical evaluation. *Radiology*, **177**, 252–257
9. Coons, H.G. (1989) Self-expanding stainless steel biliary stents. *Radiology*, **170**, 979–983
10. Adam, A., Chetty, N., Roddie, M., Yeung, E. and Benjamin, I.S. (1991) Self-expandable stainless steel endoprostheses for treatment of malignant bile duct obstruction. *AJR*, **156**, 321–325
11.. Neuhaus, H., Hagenmueller, F., Griebel, M. and Classen, M. (1991) Percutaneous cholangioscopic or transpapillary insertion of self expanding biliary stents. *Gastrointest. Endosc.*, **37**, 31–37
12. Lammer, J. (1990) Biliary endoprostheses: Plastic versus metal stents. *Radiol. Clin. North Am.*, **28**, 1211–1222
13. Cwikiel, W., Ivancev, K. and Lunderquist, A. (1990) Metallic Stents. *Radiol. Clin. North Am.*, **28**, 1203–1210
14. Mueller, P.R., Ferrucci, J.T., Teplick, S.K., van Sonnenberg, E., Haskin, P.H., Butch, R.J. and Papaniculaou, N. (1985) Biliary endoprosthesis: Analysis of complications in 113 patients. *Radiology*, **156**, 637–639
15. Lammer, J. and Neumayer, K. (1986) Biliary drainage endoprostheses: experience with 201 placements. *Radiology*, **159**, 625–629
16. Speer, A.G., Cotton, P.B., Russell, R.C.G., Mason, R.R., Hatfield, A.R.W., Leung, J.W.C., MacRae, K.D., Houghton, J. and Lennon, C.A. (1987) Randomised trial of endoscopic versus percutaneous stent insertion in malignant obstructive jaundice. *Lancet*, **i**, 57–62

*(Accepted by S. Bengmark 23 June 1992)*

